# Old and New Biomarkers in Idiopathic Recurrent Acute Pericarditis (IRAP): Prognosis and Outcomes

**DOI:** 10.1007/s11886-024-02170-y

**Published:** 2025-01-11

**Authors:** Ruggiero Mascolo, Emanuele Bizzi, Martina Martelli, Chiara Facoetti, Giulia Colazzo, Fabio Barone, Antonio Brucato

**Affiliations:** 1 Division of Internal Medicine, Fatebenefratelli Hospital, ASST Fatebenefratelli Sacco, University of Milan, Piazzale Principessa Clotilde, 3, Milan, 20121 Italy; 2 Department of Biomedical and Clinical Sciences, University of Milan, Via G.B. Grassi, 74, Milan, 20157 Italy

**Keywords:** Idiopathic recurrent acute pericarditis, Autoinflammatory disease, Inflammasome, Biomarkers, Prognosis

## Abstract

**Purpose of Review:**

To outline the latest discoveries regarding the utility and reliability of serum biomarkers in idiopathic recurrent acute pericarditis (IRAP), considering recent findings on its pathogenesis. The study highlights the predictive role of these biomarkers in potential short- (cardiac tamponade, recurrences) and long-term complications (constrictive pericarditis, death).

**Recent Findings:**

The pathogenesis of pericarditis has been better defined in recent years, focusing on the autoinflammatory pathway. New studies have demonstrated the pivotal role of the classical inflammatory biomarkers in distinguishing pericarditis phenotypes (high-grade vs. low-grade inflammation) and in defining outcomes of this condition.

**Summary:**

Pericarditis involves intense inflammatory activity, which causes elevation of different markers, such as C-reactive protein, erythrocyte sedimentation rate, neutrophils and platelets, serum amyloid A and D-Dimer. Conversely, lymphocytes are often reduced, as well as hemoglobin during the acute phase. Cardiac troponins T and I are elevated in up to 30% of cases. A Biomarker for CRP-negative cases is needed. Other markers have been proposed for diagnosis and prognosis in IRAP, such as anti-heart antibodies and anti-intercalated disk antibodies, but we need further studies to validate them.

## Introduction

Idiopathic recurrent acute pericarditis (IRAP) represents an inflammation of the pericardium layers; it represents the most frequent pericardial disease and accounts for 5% of all Emergency Room admissions for acute chest pain [1]. The aetiology of IRAP is still debated because molecular testing of pericardial fluid is not commonly performed, and standard blood tests do not detect specific causes. While viral aetiology is suspected, it has not been proven. There have been a limited number of studies on the use of cardiac Troponin I (cTnI) in diagnosing viral acute pericarditis [2], adenosine deaminase (ADA) in pericardial fluid for tubercular pericarditis [3], and classical serum tumour markers like Carcinoembryonic Antigen (CEA) for neoplastic pericarditis [4]. Idiopathic causes account for 80–90% of cases in immunocompetent patients from developed countries [5], unveiling difficulties for physicians to understand the underlying mechanism of this condition. Typical complications of this condition are recurrences, which involve 10–30% of patients with a first attack. The rate increases up to 50% after a first recurrence, particularly, if the patient was treated with steroids [6]. According to 2015 ESC guidelines, criteria to diagnose acute pericarditis include typical pericardial chest pain, pericardial friction rubs, new widespread ST-segment elevation or PR-segment depressions, and new or worsening pericardial effusion on echocardiography [7]. At least two of these criteria are present for clinical diagnosis and the elevation of inflammatory markers, mainly C-reactive protein (CRP), confirms the diagnosis [7]. Beyond diagnosis, a reliable biomarker should be able to predict outcomes and guide treatment [8]. Serum analytes could be useful in clinical practice due to their low cost and minimal invasiveness. Pericarditis involves intense inflammatory activity and elevation of markers, such as CRP, erythrocyte sedimentation rate (ESR), white blood cells and their precursors, platelets, and other acute-phase reactants, which lack specificity and predictive value. These biomarkers, along with cardiac troponins T (cTnT) and I (cTnI), D-dimer (D-D), cardiac magnetic resonance imaging (cMRI) findings, and epicardial fat volume have been studied for diagnostic and prognostic purposes [6, 9–12]. These markers are still useful tools for the diagnosis and prognosis of pericarditis. However, most studies evaluating these biomarkers were small and observational, so their findings should be interpreted cautiously. This narrative review aims to outline the latest discoveries regarding the effectiveness and reliability of serum biomarkers in IRAP, considering recent findings on its pathogenesis. The study highlights the predictive role of these biomarkers in potential short- (cardiac tamponade, recurrences) and long-term complications (constrictive pericarditis, death).

## Pathogenesis and Clinical and Laboratory Implications

Dealing with new biomarkers and their diagnostic and predictive value, we need to explore the pathogenesis of IRAP. Different pre-clinical and clinical studies discovered a potential interplay between innate and adaptive immunity in the pathophysiology of pericarditis, describing a relationship between autoimmune and autoinflammatory mechanisms. Autoimmune diseases, such as rheumatoid arthritis, systemic lupus erythematosus, Sjogren’s syndrome, Behçet’s disease, inflammatory bowel diseases and vasculitic processes may be complicated by pericardial effusion and pericarditis [13–15]. The positive response to glucocorticoids or immunosuppressants represents another important clue supporting the hypothesis of an adaptive immunity imbalance in relapsing pericarditis [16]. Different infectious (cardiotropic viruses, bacteria and other microbial hosts) and non-infectious (surgery, ischemia, irradiation, trauma, bleeding) triggers [17], interplaying with other genetic and environmental factors [18], could induce and contribute to direct or indirect damage in pericardium layers: viral infections can trigger an autoimmune response against the pericardium due to antigen mimicry. In addition, non-infectious injuries can lead to an immune response by exposing or releasing cardiac autoantigens. These processes activate B and T lymphocytes, mainly Th1 and Th17, leading to the release of interleukin (IL)-6, IL-8, and interferon (INF)-γ and possible production of anti-nuclear (ANA), anti-heart (AHA) and anti-intercalated disk (AIDA) autoantibodies, which stimulate proinflammatory damage [17, 19]. Cytokines are detectable only in pericardial fluid as a local pro-inflammatory reaction, whereas autoantibodies are checked in serum and pericardial fluid [20, 21]. A possible differential diagnosis could be made using cytokine signatures in pericardial fluid, where high tumour necrosis factor (TNF)*α* and low transforming growth factor (TGF) *β*1 levels correlate with viral pericarditis, whereas a low IL-6 concentration with autoreactive pericarditis [21]. An interesting prognostic aspect of adaptive immunity is a reduction of naive-T cells and the overexpression of activated CD8 + T effector correlate with a more active refractory inflammatory response; on the other hand, a higher number of regulatory T cells and normal expression of naive T and activated CD8 + T cells should suggest a controlled inflammatory phenotype [22]. Further studies could be useful to assess the reliability of these markers in diagnosis and prognosis.

More recently, a correlation between IRAP and autoinflammatory diseases has been speculated: a dysregulation of the inflammasome, a large intracellular multiprotein complex in neutrophils and macrophages, might overproduce pro-inflammatory cytokines such as IL-1 and TNF*α*. Recurrent pericarditis occurs in the setting of different genetic autoinflammatory diseases, particularly in tumour necrosis factor receptor-associated periodic syndrome (TRAPS), familial Mediterranean fever (FMF) and, rarely, mevalonate kinase deficiency, in which mutations in genes involved in the inflammatory response provide an overexpression of several pro-inflammatory cytokines [23]. The best-characterized inflammasome has a sensor molecule called NLR pyrin domain-containing 3 (NLRP3), activated in several inflammatory conditions, including gout, atherosclerosis and pericarditis [24–26]. The inflammasome is activated by binding of pathogen- and damage-associated molecular patterns (PAMPs and DAMPs) with specific membrane or intracellular receptors, such as Toll-like receptors (TLRs) and NOD-3 receptors, and high IL-1 concentration is released into the site of injury, with recruitment of important effector cells, mainly neutrophils, and enhancement of inflammation [27, 28]. In this context, the early stages of IRAP show a predominantly neutrophilic microenvironment, along with other immune cells and exfoliated and disintegrated mesothelial cells [16]. Neutrophils have a pivotal role in autoinflammatory diseases and IRAP [29, 30]. They can destroy cell debris and microbes through a process called phagocytosis but also release many pro-inflammatory mediators such as IL-1, IL-1 receptor antagonist, IL-6, IL-12, TGFβ, TNFα, oncostatin M and B lymphocyte stimulator (BLYSS) to activate themselves and other immune cells, mainly macrophages [29, 31]. Additionally, neutrophils create neutrophil extracellular traps (NETs), web-like structures made of cytosolic and granule proteins assembled on a scaffold of decondensed chromatin, which neutralize pathogens but, if dysregulated, they can induce the production of IL-6 and pro-IL-1β [32]. Moreover, neutrophils interact with platelets by releasing chemokines such as CC-chemokine ligand 5 (CCL-5) and platelet factor 4 (PF4); these molecules promote further neutrophil and monocyte adhesion, NETosis, and the secretion of granules, developing microthrombosis in a complex mechanism known as “thromboinflammation” [29, 31].

Table 1 summarizes the principal biomarkers in pericarditis, their utility and limitations.

**Table 1:** Summary of principal biomarkers for diagnosis and prognosis in pericarditis: utility and limitations*

Biomarker	Utility	Limitations
C-reactive protein (CRP)	• High sensitivity for diagnosis. • High levels associated with complications (cardiac tamponade, pericardiocentesis or pericardiectomy, recurrences). • Useful to monitor treatment response and tapering.	• Low specificity (infectious and non-infectious inflammatory conditions).
Leukocytes and differential formula (neutrophils, lymphocytes and NLR)	• High sensitivity for diagnosis. • Neutrophilia, lymphocytopenia and high NLR predictive of inflammatory phenotype. • High NLR predictive of recurrences and complications (cardiac tamponade). • Useful to monitor treatment response and tapering.	• Low specificity. • High neutrophilia and CRP could misdiagnose bacterial infections, inducing inappropriate antibiotic treatment. • Steroid administration could affect neutrophil count.
Hemoglobin (Hb)	• Transient normocytic anemia during an acute attack, with rapid recovery during remission. • Associated with elevated CRP levels.	• Not specific for inflammatory conditions. • Limited data available in IRAP.
D-dimer (D-D)	• Increased levels associated with increased CRP and inflammatory phenotype. • Correlated with pleuropericardial involvement. • D-D levels increase earlier than CRP.	• Low specificity with elevated value in any inflammatory condition. • Venous thromboembolism and acute aortic syndrome may be considered in proper settings.
Cardiac Troponin I (cTnI) and T (cTnT)	• Correlated with myocardial involvement. • In most cases, mild transient elevation was described. • Increased levels not predictive of adverse short- or long-term outcomes in the context of IRAP.	• Elevated cTnI (> 1.5 ng/mL) could misdiagnose ACS, in the presence of CK-MB elevation and ST-segment alteration in ECG.
INFLA-score	• Novel composite tool with different classical biomarkers. • Possibly useful to diagnose low-grade-inflammatory phenotype.	• Lack of external validation.
Antinuclear antibodies (ANA)	• Useful for autoimmune-related forms. • Higher titers associated with recurrences.	• Low specificity: they are often a clinically no specific finding.
Anti-heart (AHA) and anti-intercalated disk (AIDA) antibodies	• Possible role in autoimmune pathogenesis. • Associated with a higher number of recurrences and hospitalizations.	• Increased levels also in myocarditis and dilated cardiomyopathy. • Lack of external validation.
Serum amyloid A (SAA)	• Increased levels, with normal CRP and ESR, could indicate subclinical inflammation. • Possible marker of cardiovascular risk.	• Low specificity (inflammatory conditions and cardiovascular events). • Limited data in IRAP.

*Abbreviations: ACS, acute coronary syndrome; CK-MB, MB isoenzyme creatine kinase; ESR, erythrocyte sedimentation rate

### C-reactive Protein (CRP)

CRP is the primary and most important acute-phase protein found in the serum [33]. Its levels rise as a non-specific response to infectious and non-infectious inflammatory processes. This protein is produced only by hepatocytes and is mainly controlled by cytokines, particularly IL-6, which is released in the bloodstream during inflammation. Its serum concentration is determined only by its synthesis rate, according to the severity of the inflammatory stimulus. Once the stimulus ceases completely, the circulating CRP concentration drops rapidly, almost at the same rate as the plasma CRP clearance [34]. In a prospective study, high-sensitivity CRP (hs-CRP) was found elevated in 78% of 200 patients at the onset and during recurrences of IRAP. Among patients with normal CRP levels at 24 h from the onset of symptoms (22% of 200), 34% of them showed an increase when tested later and another 50% of them had either received anti-inflammatory treatment previously (that might impair CRP increase, particularly corticosteroids and anti-IL1 agents) or not re-evaluated afterwards. In another retrospective study on 202 patients, hs-CRP levels were elevated in 76% of patients within 6 h after onset; the percentage rose to 96.7% when retested after 24 h and 98.7% after 48 h [35]. In fact, CRP has its kinetics (as troponins, for instance): after a single stimulus, CRP concentrations in the serum increase rapidly to more than 5 mg/L in about 6 h, reaching a peak around 48 h; the plasma half-life of CRP is approximately 19 h and remains constant under all conditions of health and disease [34]. Interestingly, in acute myocarditis, CRP is often stably normal or near normal and we might speculate that CRP is lower in cases of associated myocarditis [36–38]. If the initial result is negative in suspected pericarditis, we recommend to retest for CRP after 12–24 h. Overall, 15–20% of cases of acute pericarditis have persistently normal CRP [39]. The pathogenesis of these forms with low-grade inflammation is not known. CRP is driven mainly by IL-6. However, in viral infections and autoimmune diseases characterized by the Type I interferon gene signature, CRP seems to be an unreliable marker of inflammation: in fact, the levels of CRP in the bloodstream can be relatively low, despite the presence of significant inflammation, as indicated by elevated IL-6 levels. Moreover, different CRP individual responses may be genetically regulated [40].

Pericarditis with elevated CRP usually shows clear signs of inflammation, such as fever, pericardial and pleural effusion, high white blood cell counts with neutrophilia and lymphopenia and elevated ESR; to date, pleuropulmonary involvement is highly predictive of the inflammatory phenotype [35, 39]. These patients are treated more frequently with pericardiocentesis and anti-IL1 drugs.

CRP also has a predictive value. High CRP levels in IRAP at onset are associated with major in-hospital cardiac complications, such as cardiac tamponade, non-obstructive cardiogenic shock, ventricular tachycardia, large symptomatic pericardial effusion requiring pericardiocentesis or pericardiectomy, up to death [35].

Either CRP or hs-CRP can be tested. We did not notice relevant differences between the two tests when used in patients with pericarditis. This is likely because hs-CRP is mainly used to reliably detect minimal elevations (e.g. 0.2 vs. 0.4 mg/dL), primarily associated with cardiovascular risk [41]. On the other hand, non-hs-CRP tests are useful in acute inflammatory conditions, where more pronounced elevations are observed (e.g. values higher than 1 mg/dL, often 10–20 mg/dL). In other words, for values in the high range, such as 1 mg/dl or higher, both test are reliable and reproducible. After treatment begins, it is important to monitor CRP levels to assess the effectiveness and duration of treatment with non-steroidal anti-inflammatory drugs (NSAIDs), colchicine, steroids, and anti-IL1 agents [6, 7, 42–44]. Typically, CRP levels normalize slowly (weeks) after treatment with NSAIDs [36], but quickly, in a few days, with corticosteroids and anti-IL1 drugs, usually anakinra [45] or rilonacept [44]. In most cases (85%), hs-CRP normalizes within 2 weeks of treatment. However, hs-CRP remains elevated in 40% of patients after the first week and in 15% after the second week, necessitating prolonged therapy of more than 2 weeks [36]. During the initial acute phase, we recommend regularly monitoring CRP levels on a weekly basis to track disease activity. It is important to avoid reducing the initial drugs doses too early, as this could lead to a reactivation of pericarditis while CRP levels are still high. We suggest considering tapering off the medication only when CRP levels have completely normalized.

Persistent elevation of CRP longer than 1–2 months in our opinion should be considered as a risk factor for a specific aetiology, not yet identified, or for an incessant course and recurrence, along with other factors such as fever > 38 °C, corticosteroid use and incomplete response to medical therapy [46, 47]. To gradually taper treatment, it is crucial to regularly monitor both the signs and symptoms of the disease, as well as inflammatory markers, especially CRP levels, typically on a monthly basis. Specifically, lowering the prednisone dosage should only be considered if the patient is symptom-free and their C-reactive protein level are normal [7].

### Complete Blood Cell Counts (CBC) with Differential Formula

In cases of IRAP, like in autoinflammatory diseases, white blood cells rise, with significant increases in absolute and relative numbers of neutrophils and decreases in absolute and relative numbers of lymphocytes, leading to a higher NLR [10, 39, 48]. The NLRP3-produced IL-1β induces the proliferation of granulocyte precursors (promyelocytes, myelocytes, metamyelocytes) in the bone marrow and the recruitment of neutrophils into the peripheral blood [10, 39] and pericardium [49], suggesting a direct involvement of neutrophils in the serosal tissue [29, 30]. In a study by Kim et al. in pericardial fluid, patients who developed effusive-constrictive pericarditis after pericardiocentesis showed elevated concentrations of leukocytes with a higher percentage of neutrophils and a lower percentage of monocytes [49]. Indeed, during both the first attack and recurrences, these haematological alterations correlate with a marked systemic acute-phase response, as evidenced by CRP and ESR levels, along with pleural and possible peritoneal effusion [10]. Increased CRP and neutrophil leukocytosis should not be mistakenly considered signs of bacterial infection in this context, prompting an escalation of antibiotic therapy [10, 39, 50]. To this regard, we have recently provided data from a large cohort of patients that demonstrated that pericarditis with a systemic inflammatory phenotype is often a manifestation of a systemic autoinflammatory disease, most frequently with a pleuropulmonary involvement and that this specific pericarditis phenotype shows similarities with other autoinflammatory diseases [39]. To date, IRAP shares clinical and laboratory similarities with the inflammatory syndromes where, beyond periodic fever, increased CRP and polyserositis, high NLR have been demonstrated [48, 51, 52].

Counts of specific types of white blood cells, particularly neutrophils and lymphocytes, as well as the neutrophil-to-lymphocyte ratio (NLR), can serve as valuable prognostic markers for various medical conditions, including stroke [53, 54], acute myocardial infarction (AMI) [55, 56], sepsis [57].

Peripheral blood differential counts have a predictive value in IRAP: relative neutrophilia, absolute and relative lymphopenia with high NLR can indicate a higher risk of developing 12-month recurrences [10]; the NLR and CRP are also independent predictors of 12-month recurrences and cardiac tamponade [58]. Lastly, as CRP levels, complete blood cell count with differential formula may be used to monitor the response of treatment.

Immature granulocytes, which measure the number of promyelocytes, myelocytes, and metamyelocytes in peripheral blood, can serve as a novel biomarker for increased bone marrow activation and inflammation [59]. It could be an indicator of severity and mortality in different acute conditions such as sepsis [60], acute pancreatitis [61], upper gastrointestinal bleeding [62] and coronary artery diseases (CAD) [63]. Selvi et al. found a high percentage of immature granulocytes in patients with acute pericarditis. A value higher than 65% has high sensitivity (80%) and specificity (90.6%) levels [9].

Another blood cell type involved in the inflammatory process, platelets, is not well defined in IRAP. Further research should be implemented to define their role in IRAP. In our clinical experience, hemoglobin levels may decline rapidly during IRAP, with transient normocytic anemia, followed by a rapid rebound at remission, with a median hemoglobin reduction of 1.4 g/dL. Hemoglobin reduction was associated with CRP elevation [64]. In this regard, IRAP may represent a pathogenetic model for this type of anemia, mediated by hepcidin activity [65].

### D-Dimer (D-D)

D-dimer (D-D) is a specific product of fibrin degradation. Increased levels of this biomarker may occur in patients with different thrombotic disorders [66]. Despite the low specificity [67] and interference of various physiological and pathological factors [66], D-D testing is currently common in the emergency department to rule out life-threatening conditions such as pulmonary embolism or acute aortic syndrome [68–70]. Moreover, D-D can provide insights into other clinical conditions, including disseminated intravascular coagulation, acute coronary syndromes (ACS) and ischemic stroke [71]. D-D elevation was found in a significant proportion (50.6 to 73.3%) of patients diagnosed with acute pericarditis [12, 72]; increased values correlated with fever [72], higher CRP concentration [12, 72, 73], neutrophilic leukocytosis [72], lower haematocrit, higher platelet count [12, 74] and hospitalization [12]. Studies also described a higher rate of pleural and pericardial effusions in patients with high D-D levels during the first attack, with a trend toward cardiac tamponade [12, 72, 75]; in patients with pericardial effusion following coronavirus disease, Begic et al. showed a correlation between DD and CRP levels and pericardial effusion, respectively around the right atrium and next to the right ventricle-free wall; they proposed that these findings could indicate endothelial dysfunction and microthrombosis of the pulmonary circulation [76]. These aspects suggest that D-D could be considered a measure of the inflammatory burden, based on the complex interplay between inflammation and coagulation in venous thrombogenesis [71, 77]. D-D and CRP may exhibit different kinetics, with D-D rising earlier, as observed in post-operative infections [78].

### Cardiac Troponin T (cTnT) and I (cTnI)

Beyond CAD, some studies have identified elevated levels of cardiac troponins in various clinical conditions, including congestive heart failure, myocarditis, amyloidosis and pulmonary embolism [69]. Elevation of cardiac troponins may also occur in cases of acute pericarditis [2, 7, 79, 80]. Notably, up to 32.2% of patients with IRAP exhibit some degree of elevation in cardiac-specific troponin I (cTnI) or T (cTnT) [2, 81]. The concentrations and kinetics of these elevations differ from those observed in CAD and myocarditis, suggesting that inflammation, rather than myocyte death, is responsible for the concurrent involvement of the subepicardial myocardium [79, 82]. Two distinct types of myocardial involvement can be identified: myopericarditis and perimyocarditis. Patients with myopericarditis typically show elevated serum levels of cTnT or cTnI, as well as myocardial involvement evident on cMRI, without any new abnormalities in focal or global left ventricular function [6, 7]. In this context, the degree of troponin elevation appears to correlate with the level of inflammatory activity, as observed in myocarditis [83, 84]; the extent of ST-segment elevation is associated with inflammatory severity, and troponin levels usually normalize within one to two weeks after diagnosis [1]. Notably, elevated troponin concentrations are more common in younger male patients, with pericardial effusion at onset [2, 79, 81, 85], due to important inflammatory activity [79]. In some cases, a rise in troponin levels could misdiagnose an acute coronary syndrome (ACS), in particular with high sensitivity measurement. In a retrospective study, high sensitivity (hs)-cTnI was elevated in 34 out of 69 hospitalized patients; among these, 15 (22%) had concentrations beyond the threshold for AMI which is 1.5 ng/mL [79]. In another study involving 14 patients in the Emergency Room, 10 patients showed concentrations and kinetics of cTnI similar to those observed in ACS [80]. To date, two different temporal patterns of cTnI release are recognized: most frequently, a mild increase of serum cTnI without MB isoenzyme creatine kinase (CK-MB) elevation, lasting for 3 days; less frequently, a higher increase of serum cTnI, together with CK-MB elevation, echocardiographic wall motion abnormalities, and a prolonged pattern similar to that observed in AMI, due to more severe myocardial involvement [2]. To diagnose myopericarditis, CAD must be excluded by coronary angiography and/or cMRI. Conversely, perimyocarditis refers to the inflammatory involvement of the pericardium and myocardium, characterized by new onset or worsening of focal or diffuse left ventricular wall motion abnormalities [6, 7]. Increased troponin levels in cases of IRAP do not predict adverse short- or long-term outcomes [2, 79, 81]: unlike ACS, elevated levels of troponins are not a negative prognostic marker in this setting [47, 82, 86].

### Composite Biomarkers: INFLA-Score

CRP may be inadequate in evaluating pericarditis with low-grade inflammation [34, 87] and may be normal in 15–20% of IRAP patients [36, 39, 88]. Subclinical inflammation is a risk factor for various chronic diseases, including cardiovascular events. The INFLA-score is an algorithm proposed for assessing low-grade inflammation and related cardiovascular risk. It utilizes CRP, white blood cell and platelet counts, and neutrophil-to-lymphocyte ratio. Each parameter is assigned a value ranging from − 16 to + 16 and the total sum defines the score. A score greater than 0 indicates the presence of subclinical inflammation [89]. Considering the pathogenesis of idiopathic pericarditis, Andreis et al. reported using INFLA-score in diagnosing pericarditis. The INFLA-score can detect low-grade inflammatory pericarditis, especially in patients with normal CRP, with high specificity (97%) and positive likelihood ratio (13), using a cutoff of 10, and high sensitivity (86%) and low negative likelihood ratio (0.22) when a cutoff of 0. Moreover, a positive INFLA-score defines a high risk of 6-month recurrences [90]. Further studies are needed for external validation and the reliability of this score for diagnosis and prognosis in IRAP.

### Other Biomarkers

Antinuclear antibodies (ANA) are positive in several systemic and organ-specific autoimmune diseases and chronic infections [91]. Notably, about 5 to 10% of healthy individuals could have a low level of antibodies and could be associated with various medications [92]. ANA positivity is not specific and clinical relevance is related to the clinical context. ANA are more prevalent among women, older individuals and those of African/American descent [91, 93, 94]. When portrayed correctly, they may provide valuable diagnostic and prognostic information [95]. Based on the autoimmune pathway, ANA have been detected in 43.4% of patients with IRAP, compared with 9.8% of controls. Most of them have low titers (1/40–1/80), while moderate titers (1/160–1/320) can be found in 26.7% of cases [96]. Brucato found that 59% of patients with IRAP had an ANA titer exceeding 1/80, and 15% had a titer of 1/160, which was associated with a high risk of recurrence [97]. Additionally, approximately 15% of cases tested positive for Rheumatoid factor, and 8% of the patients tested positive for anti-Ro/SSA [97]. Overall, the ANA positivity in IRAP patients does not modify the management; during a mean follow-up of 32 months, complications and new rheumatological diagnoses were similar in patients with and without ANA positivity [96].

Anti-heart (AHA) and anti-intercalated-disk antibodies (AIDA) are found in the serum of patients with biopsy-proven myocarditis [98] and dilated cardiomyopathy [99]. AHA recognizes ɑ and β myosin heavy chain and myosin light chain-1v isoforms [100] while the autoantigens responsible for AIDA are not identified; in these conditions, they may play a role in the autoimmune pathogenesis, causing cytolysis in vitro of cardiomyocytes and correlating their levels with cytolytic serum activity. AHA and/or AIDA were found in 67.5% of patients with IRAP, with a higher frequency of cross-reactive 1 AHA and AIDA (respectively, 50 and 25%) than in other non-inflammatory cardiac diseases (5%, 6%) or normal subjects (3%, 0%), respectively [20]. Different AHA antibody specificities may be associated with pericarditis and myocarditis, with cross-reactive AHA more prevalent in pericarditis, while organ-specific AHA in myocarditis [101]. In IRAP, AIDA positivity was associated with a higher number of recurrences and hospitalizations, whereas AHA positivity with a longer duration of symptoms and a higher number of recurrences [20]. Future research is required to define the reliability and clinical impact of these autoantibodies.

Serum amyloid A (SAA) represents a major acute-phase reactant [33] in different clinical conditions, such as rheumatoid arthritis, cardiovascular diseases, solid cancer, and infections [102–106]. Increased serum levels along with normal CRP and ESR levels indicate subclinical inflammation [107]. Studies have suggested that high levels of SAA may be useful in predicting cardiovascular events [102, 103, 108]. As in FMF [107, 109], SAA can be a valuable IRAP biomarker. Its levels increase during IRAP recurrences and may be slightly elevated even during symptom-free periods [110]. Kaudewitz found that SAA levels were lower in IRAP than in post-cardiac injury pericarditis and correlated with a higher risk of recurrences [111]. Further studies are needed to explore deeply the diagnostic and prognostic role of this marker in IRAP.

Procalcitonin has been tested in 50 IRAP patients, with a strong inflammatory phenotype, with only 4 showing marginal elevation [72]; these findings reinforce the inflammasome-mediated pathogenesis and the avoidance of escalation in antibiotic therapy [39].

Other potential markers evaluated in IRAP are adipokines [110], possibly correlated with increased cardiovascular risk [112–114], MHC class I polypeptide–related sequence A (MICA), CEA Cell Adhesion Molecule (CEACAM1) [115] and microRNA (miR) [116]. Further studies are warranted.

**Fig. 1:**
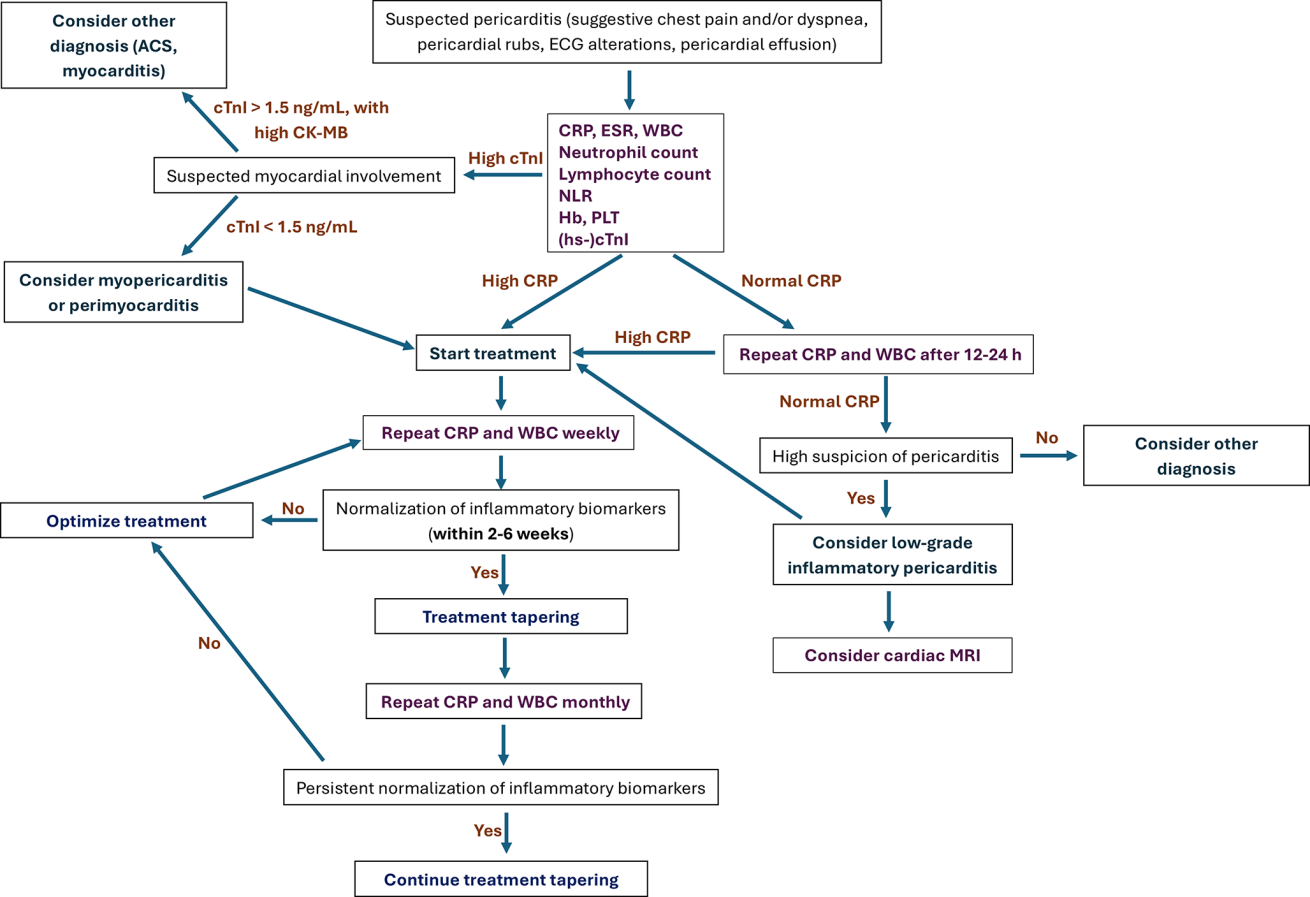
Proposed algorithm for the possible use of biomarkers in recurrent pericarditis. Abbreviations: ACS, acute coronary syndrome; ECG, electrocardiogram; CRP, C-reactive protein; ESR, erythrocyte sedimentation rate; WBC, white blood cells; NLR, neutrophil-to-lymphocyte ratio; Hb, hemoglobin; PLT, platelets; (hs-)cTnI, (high sensitivity-) cardiac troponin I; MRI, magnetic resonance imaging.

Figure 1 shows a proposed algorithm that describes the possible use of biomarkers in IRAP.

## Conclusions

Since the pathogenesis of pericarditis has been better defined in recent years, new studies have demonstrated the pivotal role of the classical biomarkers in IRAP. CRP, CBC with differential formula, D-D and SAA represent the most important inflammatory biomarkers in IRAP. Elevated levels are useful to confirm the diagnosis but are also important for predicting the outcomes of IRAP (recurrences and tamponade). However, CRP-negative pericarditis is an interesting phenotype of IRAP which lacks such clear inflammatory characteristics, making it challenging to investigate and manage. Despite novel insights into pathogenesis in recent years, biomarkers for CRP-negative pericarditis, such as SAA or IL-6, are still an unmet need and this can be an intriguing area of research, considering the possible role of IFN gene signature and the possible genetic regulation of CRP response in other immunological conditions, such as SLE.

Troponins are markers of myocardial involvement in IRAP, and notably elevated levels of troponins do not have a negative prognostic value in IRAP; this is possibly explained by the fact the concomitant myocardial involvement in the context of pericarditis is probably related to a viral aetiology, a condition that is probably more benign than other secondary forms.

The almost universal negativity of procalcitonin, even in cases with very high levels of CRP, fever and neutrophil leucocytosis, is consistent with the absence of underlying bacterial infections, helping clinicians in avoiding antibiotics escalation.

ANA, AHA and AIDA are biomarkers that may be related to the pathogenesis of this condition, with AHA and AIDA possibly related to outcomes, while ANA is not.

The therapy with IL-1-pathway inhibitors is very effective in selected cases of IRAP; evidence is increasing that also therapy with these agents is often long-lasting [42, 117]. Unfortunately, we do not have so far a reliable biomarker that can predict when to stop anti-IL1 agents [118].

## Key References

- Tombetti, E. et al. Relapsing pericarditis: Peripheral blood neutrophilia, lymphopenia and high neutrophil-to-lymphocyte ratio herald acute attacks, high-grade inflammation, multiserosal involvement, and predict multiple recurrences. *Int J Rheum Dis* 26, 337–343 (2023).
-
  - This study evaluated diagnostic and predictive role in IRAP of classical inflammatory biomarkers (white blood cells with differential formula). Neutrophilia, lymphopenia and NLR represent typical features of IRAP.
- Lazaros, G. et al. D-dimer as a diagnostic and prognostic plasma biomarker in patients with a first episode of acute pericarditis. *Eur J Intern Med***116**, 58–64 (2023).
-
  - Results of this study demonstrated the diagnostic and prognostic role of D-dimer.
- Pisacreta, A. M. et al. Acute pericarditis with pleuropulmonary involvement, fever and elevated C-reactive protein: A systemic autoinflammatory disease? A cohort study. *Eur J Intern Med* 113, 45–48 (2023).
-
  - This paper reported how recurrent pericarditis can be an epiphenomenon of a systemic inflammatory disease according to clinical, laboratory and prognostic features.

## Data Availability

No datasets were generated or analysed during the current study.
